# Effects of dietary phosphorus and *myo*-inositol supplementation on NaPi-IIb and TRPV6 protein expression in duodenal apical membranes of laying hens from two strains

**DOI:** 10.1016/j.psj.2025.106171

**Published:** 2025-11-30

**Authors:** Nataliia Shomina, Vera Sommerfeld, Anna Hanauska, Michael Oster, Markus Rodehutscord, Korinna Huber

**Affiliations:** a Institute of Animal Science, University of Hohenheim, Stuttgart, Germany; b Research Institute for Farm Animal Biology (FBN), Dummerstorf, Germany

**Keywords:** Mineral phosphorus, *Myo*-inositol, Intestinal transporter, Laying hen

## Abstract

Phosphorus (**P**) and calcium (**Ca**) absorption in the intestine is mediated by apical brush border membrane (**BBM**) transporters, including the sodium-dependent phosphate (**P_i_**) transporter NaPi-IIb and the Ca²⁺-selective channel TRPV6. Both are highly expressed in the duodenum and exhibit dietary adaptability; yet little is known about how this adaptability varies with strain and age in laying hens. The present study examined the effects of dietary mineral P renunciation and *myo*-inositol (**MI**) supplementation on NaPi-IIb and TRPV6 protein expression in the duodenal BBM of Lohmann Brown-Classic (**LB**) and Lohmann LSL-Classic (**LSL**) hens. Two independent feeding trials were conducted: hens received diets either with or without mineral P supplementation (wk 15 - 19 and 20 – 24), or with graded MI levels (0 - 3 g/kg; wk 26 – 30). At the end of each period, hens were euthanized and protein expression of NaPi-IIb and TRPV6 in duodenal BBM was studied by western blotting. Statistical correlation with additional traits of mineral metabolism was analyzed. An immunoreactive NaPi-IIb band was detected at ∼ 45 kDa; therefore, all results reported here refer to this NaPi-IIb fragment. Mineral P renunciation did not affect NaPi-IIb fragment or TRPV6 expression in either hen strain. In LSL hens NaPi-IIb fragment expression increased from wk 19 to wk 24, whereas in LB hens it remained unchanged. NaPi-IIb fragment expression was positively associated with duodenal phosphatase activity and plasma estradiol. TRPV6 expression tended to reduce in LB hens from wk 19 to wk 24, but remained stable in LSL hens. TRPV6 expression was positively associated with duodeno-jejunal P content. MI supplementation upregulated NaPi-IIb fragment expression in LB, but downregulated it in LSL hens with high dietary MI levels, without affecting TRPV6. These findings demonstrate strain-dependent regulatory patterns of duodenal expression of NaPi-IIb fragment and TRPV6 in response to physiological stage and MI supply, indicating that mineral feeding strategies may benefit from genotype-specific consideration, whereas the mechanisms underlying MI-related effects require further clarification.

## Introduction

Phosphorus (**P**) and calcium (**Ca**) are two essential minerals that play fundamental roles in skeletal development, eggshell formation, and metabolic regulation in laying hens. Maintaining an optimal balance between these minerals is crucial, as excess dietary P increases its excretion, contributing to environmental pollution (Sharpley, 1999), whereas an inadequate supply may compromise skeletal health (Kebreab et al., 2009). To prevent deficiency, commercial layer diets are commonly supplemented with inorganic mineral P sources (Jing et al., 2018). However, recent evidence indicated that the actual P requirement of laying hens is lower than previously assumed (Rodehutscord et al., 2023) and that, under dietary mineral P deficiency, hens can improve the utilization of plant-based P through enhanced phytate (**InsP_6_**) hydrolysis (Sommerfeld et al., 2020b; Sommerfeld et al., 2024), thereby lowering P excretion.

In poultry, the duodenum represents the principal site for active, transcellular absorption of P and Ca (San et al., 2021). Phosphate (**P_i_**) entry into enterocytes is mediated by NaPi-IIb (encoded by *SLC34A2*), the main sodium-dependent P_i_ transporter in chicken intestine (Yan et al., 2007). It facilitates the secondary active P_i_ transport by co-transporting it with Na⁺ ions, using the inward sodium gradient established by the basolateral Na⁺/K⁺-ATPase (Yan et al., 2007). NaPi-IIb expression is highest in the duodenum, decreases along the intestinal axis, and increases under dietary P restriction (Fang et al., 2012). In turn, Ca absorption occurs mainly via the transient receptor potential vanilloid channel TRPV6, which mediates apical Ca²⁺ entry and represents a rate-limiting step in transcellular Ca transport (Hoenderop et al., 2005; Yang et al., 2011).

Regulation of NaPi-IIb and TRPV6 at the mRNA level was extensively studied in chickens, demonstrating sensitivity to luminal P and Ca availability and endocrine control (Proszkowiec-Weglarz and Angel, 2013; Garcia-Mejia et al., 2024). However, information on NaPi-IIb protein regulation in response to dietary challenges in commercial laying hens remains limited, and only a few studies reported TRPV6 protein expression in the chicken intestine (Yang et al., 2011; Huber et al., 2015). Moreover, under dietary P reduction, transcriptional responses did not necessarily correspond to protein abundance (Li et al., 2018; Meier et al., 2025), indicating that post-transcriptional regulation may play an important role in intestinal adaptation to fluctuating mineral supply.

Phytate degradation not only releases P_i_ but also liberates *myo*-inositol (**MI**), which may act as an additional modulator of P and Ca absorption processes. Beyond its nutritional role, MI serves as a precursor for inositol phosphates (**InsP**) and inositol pyrophosphates (**PP-InsPs**), such as InsP_7_ and InsP_8_, which are recognized as central intracellular messengers in P_i_ sensing and homeostasis (Su et al., 2023; Gu et al., 2024). Experimental evidence indicated that MI supplementation can increase transcript abundance of NaPi-IIb in porcine intestinal epithelial cells (Ogunribido et al., 2022), stimulate the expression of intestinal alkaline phosphatase and potentially affect Ca metabolism in laying hens (Herwig et al., 2021), suggesting potential interactions with both P_i_ and Ca transport. However, the direct regulatory role of MI in avian NaPi-IIb and TRPV6 expression remains unclear.

Recently, an extensive study was conducted comparing the responses of two commercial laying hen strains, Lohmann Brown-Classic (**LB**) and Lohmann LSL-Classic (**LSL**), to dietary mineral P renunciation and MI supplementation, revealing distinct strain-specific intestinal and systemic adaptations to dietary challenges across key physiological stages: pre-laying, onset of lay, and peak laying (Sommerfeld et al., 2024; Sommerfeld et al., 2025; Qasir et al., 2025). The present study aimed to complement these findings by characterizing the expression of NaPi-IIb and TRPV6 in the duodenal brush border membrane (**BBM**) of the same hens, offering a protein-level assessment of transcellular P_i_ and Ca transport potential. Understanding how transporter abundance varies with diet, strain, and physiological stage can inform the development of precision-nutrition strategies aimed at reducing dietary mineral P use and mitigating environmental impacts.

## Materials and methods

This study was a part of the interdisciplinary Research Unit P-Fowl: Inositol phosphates and *myo*-inositol in the domestic fowl: Exploring the interface of genetics, physiology, microbiome, and nutrition (https://p-fowl.uni-hohenheim.de/). It complements and extends recent studies within this framework (Sommerfeld et al., 2024; Abitew et al., 2024; Sommerfeld et al., 2025; Hanauska et al., 2025; Qasir et al., 2025). The animal experiments were approved by the Regierungspräsidium Tübingen, Germany (Project no. HOH67-21TE) and conducted in accordance with German animal welfare legislation. The samples used in this study originated from two animal trials, here referred to as mineral P renunciation trial and MI supplementation trial, which are fully described by Sommerfeld et al. (2024; 2025) and briefly outlined here.

### Experimental design and diets

A total of 40 LB and 40 LSL laying hens (Lohmann Breeders GmbH, Cuxhaven, Germany) were used in each trial. Hens were individually housed in metabolic units at certain age periods for 4 weeks (wk 15-19 (n = 40) and wk 20-24 (n = 40) for the mineral P renunciation trial and wk 26-30 (n = 80) for the MI supplementation trial) being fed respective experimental diets until slaughter. Treatments were arranged in randomized complete block designs. The metabolic units were equipped with a wooden perch, nest, feeding trough, water cups, and wire mesh floor.

***Mineral P Renunciation*.** The trial followed a 2 × 2 × 2 factorial arrangement of treatments, with treatments based on three factors: hen strain (LB, LSL), period (wk 19 and wk 24), and dietary mineral P supplementation (0 g P/kg feed (P-), 1 g P/kg feed (P+), supplied as monocalcium phosphate). The diets were based on corn and soybean meal to minimize the intrinsic plant phytase activity and were calculated to contain 3.1 or 4.1 g P/kg, 1.3 or 2.3 g non-phytate P (nPP)/kg (P- and P+ respectively), and 35 g Ca/kg (Sommerfeld et al., 2024).

***Myo-inositol Supplementation*.** The MI supplementation trial followed a 2 × 4 factorial arrangement of treatments based on hen strain (LB, LSL) and 4 dietary MI supplementation levels: 0 g/kg (MI0)**,** 1 g MI/kg (MI1), 2 g MI/kg (MI2), or 3 g MI/kg (MI3). The diets were based on corn and soybean meal to minimize the intrinsic plant phytase activity and were calculated to contain 2.2 g nPP/kg and 35 g Ca/kg (Sommerfeld et al., 2025).

### Slaughter and sampling

Before slaughtering in wk 19, wk 24 in mineral P renunciation trial, and wk 30 in MI supplementation trial, the feed was deprived for 1 h, followed by 1 h ad libitum access to standardize gut fill for each hen. The hens were individually stunned with a gas mixture of 35% CO_2_, 35 % N_2_, and 30 % O_2_ and killed by exsanguination.

Immediately after slaughter, trunk blood was collected, centrifuged, and the resulting plasma was stored at - 80 °C until further analysis. Relevant phenotypes related to mineral homeostasis based on these plasma samples have been described elsewhere (Sommerfeld et al., 2024; Sommerfeld et al., 2025; Qasir et al., 2025; Szentgyörgyi et al., 2025).

The duodenum was opened longitudinally and gently rinsed with ice-cold physiological saline solution (0.9 % NaCl) to remove chyme residuals. Mucosal samples from the whole section were scraped on an ice-cooled glass plate with two microscopic slides, and the scraped mucosa was immediately frozen in liquid nitrogen and stored at - 80°C until further processing (Hanauska et al. 2025; Sommerfeld et al., 2025).

### Preparation of BBM

The preparation of BBM was conducted according to Huber et al. (2015) with slight modifications described by Hanauska et al. (2025). In short, grinding mucosa samples (about 500 mg) in liquid nitrogen was followed by tissue homogenization and mixing with HEPES/mannitol buffer (HEPES 2 mM, mannitol 50 mM, PMSF 25 mM) in a glass potter with the appropriate homogenizer (homogen^plus^, shuett-biotec GmbH, Göttingen, Germany). Homogenates were incubated on ice for 20 min and 80 rpm shaking (MS Rocking Shaker, MS-NRK-30, Major Science Co., Ltd, Taiwan) after adding MgCl_2_ solution (1 mol/L) for basolateral membrane precipitation followed by centrifugation for 20 min at 3,000 × *g* (Sorvall LYNX 4000 Superspeed-Centrifuge, Thermo Scientific™, Life Technologies GmbH, Darmstadt, Germany). Precipitation of basolateral membranes by MgCl_2_ was done twice. BBM were concentrated into a pellet with centrifugation at 30,000 × *g* for 30 min, which was finally resuspended in HEPES/mannitol buffer (HEPES 20 mM, mannitol 300 mM, MgSO_4_ 0.1 mM) containing protease inhibitors (cOmplete™ Mini Protease Inhibitor Cocktail, Roche Diagnostics GmbH, Mannheim, Germany). Aliquots of the final BBM preparations were shock-frozen in liquid nitrogen and stored at -80°C until analysis. Protein content of BBM preparations was determined in triplicate by using a commercial Coomassie blue protein assay (Bradford Reagent, 5 x, SERVA, Heidelberg, Germany).

### Detection of transporter proteins by western blotting

A Western Blot using BBM was performed as previously described by Huber et al. (2015). Briefly, after heat-denaturation at 95 °C for 5 min in loading buffer (50 mM TrisHCl (pH 6.8), 10 % glycerol, 2 % sodium dodecylsulfate, and 0.25 % bromphenol blue, 2 % mercaptoethanol), 40 *µ*g of BBM protein per lane were separated by SDS-PAGE on a 5% stacking/8.1% running gels and transferred to nitrocellulose membranes using BioRad Trans Blot Turbo system (BioRad laboratories GmbH, Munich, Germany). Prepared membranes were blocked in PBS solution, containing 5% fat-free milk powder (Merck KGaA, Darmstadt, Germany) and 0.1% Tween (Carl Roth GmbH, Karlsruhe, Germany) for 120 min, and incubated overnight at 4°C with primary antibodies against NaPi-IIb, 1:1,000 (N0035-26C, US Biological, Swampscott, MA) and TRPV6, 1:2,000 (ACC-036, Alomone Labs, Jerusalem, Israel). Detection was performed using 1:2,000 anti-rabbit HRP-conjugated secondary antibodies (7074P2, Cell Signaling Technology, Danvers, MA) and chemiluminescent substrate (WesternBright^TM^ Quantum, Biozym Scientific GmbH, Germany) and imaged with the ChemiDoc^TM^ Touch Imaging System (BioRad laboratories GmbH, Munich, Germany). Protein expression was semiquantified by densitometry (ImageLab Software, version 6.0; BioRad laboratories GmbH). Finally, all membranes were stained with Indian ink (Pelican, Germany) for 30 min, washed with distilled water, quantified with colorimetric detection and used to normalize the signal intensities to the total protein loaded per band.

The specificity of NaPi-IIb antibodies was confirmed by pre-incubation with antigenic peptide. For the blocking assay, NaPi-IIb antibodies (1:30) were preincubated with either NaPi-IIb control peptide (N0035-26B, US Biological, Salem, MA) or PBS at 37°C for 120 min, then at 4°C overnight. Immune complexes were pelleted by centrifugation (10,000–15,000 rpm, 15 min, 4°C), and the supernatant was transferred to fresh tubes, diluted in 5% fat-free milk/PBS with 0.1% Tween, and used for immunodetection of NaPi-IIb proteins in BBM. The specificity of the TRPV6 antibody was not directly confirmed in this study; however, we used the same antibody that was previously employed and validated using peptide blocking assays in studies on chickens by Yang et al. (2011) and Huber et al. (2015).

### Statistical analysis

The statistical evaluations were performed using the MIXED procedure and pairwise t-tests using the SAS software package (version 9.3; SAS Institute Inc., Cary, North Carolina, USA). Normal distribution and homogeneous variances of residuals were assessed graphically in SAS. The individual hen was considered the experimental unit.

The following model was used for mineral P renunciation trial:

Y_ijklm_ = μ + α_i_ + β_j_ + γ_k_ + (αβ)_ij_ + (αγ)_ik_ + (βγ)_jk_ + (αβγ)_ijk_ + δ_l_ + ϕ_m_ + ε_ijklm_,

where Y_ijklm_ = response variable, μ = overall mean, α_i_ = effect of strain (fixed), β_j_ = effect of hen production period (fixed), γ_k_ = effect of mineral P supplementation, all 2- and 3-fold-interactions among strain, production period, and mineral P supplementation (fixed), δ_l_ = block (random), ϕ_m_ = father/rooster (random), and ε_ijklm_ = residual error (Sommerfeld et al., 2024).

The following model was used for trial MI supplementation:

Y_ijkl_ = μ + α_i_ + β_j_ + (αβ)_ij_ + γ_k_ + ϕ_l_ + ε_ijkl_,

where Y_ijkl_ = response variable, μ = overall mean, α_i_ = effect of strain (fixed), β_j_ = effect of MI supplementation (fixed), the interaction between strain and MI supplementation (fixed), γ_k_ = block (random), ϕ_l_ = father/rooster (random), and ε_ijkl_ = residual error (Sommerfeld et al., 2025).

Pearson correlation analysis was performed using the correlation matrix function in GraphPad Prism (version 9.5.1 for Windows, GraphPad Software, LLC) to assess potential relationships between transporter expression (NaPi-IIb, TRPV6) in the duodenal BBM and plasma concentrations of P_i_, Ca, MI, 25-hydroxycholecalciferol (**calcidiol**), 1,25-dihydroxycholecalciferol (**calcitriol**), parathyroid hormone (**PTH**), estradiol, concentration of P, Ca, MI, InsP_6_ in duodenum+jejunum, and duodenal phosphatase activity. These parameters were not measured directly in the present study but were obtained from the same individual hens, and several of them were reported previously (Sommerfeld et al., 2024; Sommerfeld et al., 2025; Qasir et al., 2025).

Statistical significance for all tests was set at *P* < 0.050, and trends were accepted at *P* < 0.100.

## Results

### Specificity of NaPi-IIb and TRPV6 detection in BBM of duodenal enterocytes

For NaPi-IIb, a distinct band was observed at approximately ∼ 45 kDa. Pre-incubation of the antibodies with their immunizing peptide completely abolished the ∼ 45 kDa band, confirming that it corresponded specifically to NaPi-IIb protein (Supplementary Fig. S1). As the detected molecular weight was below that of the predicted full-length NaPi-IIb, all analyses reported hereafter refer to the NaPi-IIb immunoreactive band at ∼ 45 kDa (NaPi-IIb fragment). For TRPV6, a band was detected at approximately ∼ 100 kDa, corresponding to the expected molecular weight of the full-length TRPV6 protein.

### Expression of NaPi-IIb fragment and TRPV6 in the duodenum of laying hens at two periods in response to dietary mineral P renunciation

**Fig. 1:**
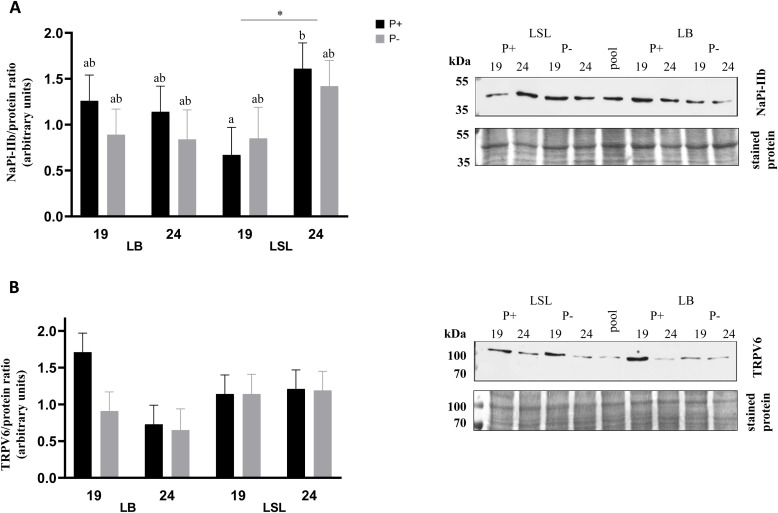
Protein expression of NaPi-IIb fragment and the transient receptor potential cation channel TRPV6 in duodenal brush border membranes (**BBM**) of laying hens (Mineral P renunciation trial). (A) The expression of NaPi-IIb immunoreactive band at ∼ 45 kDa and (B) TRPV6 at ∼ 100 kDa in duodenal BBM of Lohmann Brown-Classic (**LB**) and Lohmann LSL- Classic (**LSL**) hens before (wk 19) and after (wk 24) onset of lay and fed with (1 g/kg; P+) or without (0 g/kg; P-) supplemental mineral phosphorus. LSmeans ± SEM (n = 10) of NaPi-IIb (∼ 45 kDa) and TRPV6 (∼ 100 kDa) band intensities, expressed as arbitrary units relative to total band protein used as the loading control, are shown together with representative Western blots. Different superscripts indicate statistical differences between the experimental groups; * P < 0.050 indicate significant difference between 2 periods (wk 19 and wk 24).

Among the main effects (period, strain, and diet) and their two- and three-way interactions, only the period × strain interaction was statistically significant for the expression of NaPi-IIb fragment (*P* = 0.040) and showed a trend for TRPV6 (*P* = 0.074), whereas all other effects were not significant (*P* > 0.050; Fig. 1). In LSL hens, NaPi-IIb fragment expression increased from wk 19 to wk 24 (*P* = 0.012; Fig. 1A), while in LB hens it remained unchanged across both periods (*P* = 0.767). Post-hoc comparisons showed a higher TRPV6 expression in LB hens at wk 19 than at wk 24, whereas it remained stable in LSL hens (Fig. 1B). At wk 24, both the expression of NaPi-IIb fragment and TRPV6 tended to be higher in LSL than in LB hens (NaPi-IIb fragment: *P* = 0.082; TRPV6: *P* = 0.061), with no differences at wk 19 (NaPi-IIb fragment: *P* = 0.305; TRPV6: *P* = 0.518).

### Expression of NaPi-IIb fragment and TRPV6 in the duodenum of laying hens in response to dietary MI supplementation

**Fig. 2:**
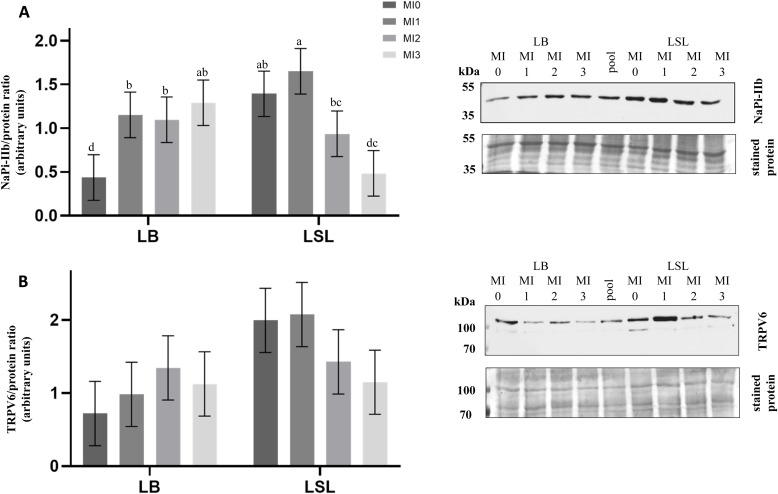
Protein expression of NaPi-IIb fragment and the transient receptor potential cation channel TRPV6 in duodenal brush border membranes (**BBM**) of laying hens (*Myo*-inositol (**MI**) supplementation trial). (A) The expression of NaPi-IIb immunoreactive band at ∼ 45 kDa and (B) TRPV6 at ∼ 100 kDa in duodenal BBM of Lohmann Brown-Classic (**LB**) and Lohmann LSL- Classic (**LSL**) hens fed diets containing 0, 1, 2, or 3 g MI/kg feed (MI0, MI1, MI2, MI3 groups respectively). LSmeans ± SEM (n = 10) of NaPi-IIb (∼ 45 kDa) and TRPV6 (∼ 100 kDa) band intensities, expressed as arbitrary units relative to total band protein used as the loading control, are shown together with representative Western blots. Different superscripts indicate statistical differences between the experimental groups.

A highly significant strain × diet interaction was observed only for the expression of NaPi-IIb fragment (NaPi-IIb fragment: *P* < 0.001; TRPV6: *P* = 0.163; Fig. 2). Regarding main effects, diet significantly affected NaPi-IIb fragment expression (NaPi-IIb fragment: *P* = 0.016; TRPV6: *P* = 0.719), while strain had no independent effect on the expression of NaPi-IIb fragment, but showed a tendency for TRPV6 (NaPi-IIb fragment: *P* = 0.326; TRPV6: *P* = 0.076). In LB hens, the expression of NaPi-fragment in duodenal BBM increased in response to MI supplementation (*P* < 0.010); although expression levels did not differ among MI1, MI2, and MI3 groups (*P* > 0.050; Fig. 2A). In contrast, in LSL hens, the expression of NaPi-IIb fragment was higher in MI0 compared to MI3 (*P* < 0.001) and in MI1 compared to MI2 and MI3 (*P* < 0.010; Fig 2A). TRPV6 expression tended to be higher in LSL hens than LB hens (Fig. 2B).

### Correlations of the NaPi-IIb fragment and TRPV6 expression levels with various systemic, endocrine, and local factors in laying hens

In the mineral P renunciation trial and when analyzed for both strains together, the expression of NaPi-IIb fragment was positively correlated with duodenal phosphatase activity (r = 0.36, *P* = 0.001) and plasma estradiol levels (r = 0.25, *P* = 0.034), while it was negatively associated with PTH (r = - 0.29, *P* = 0.014; Table 1). These correlations were more pronounced in LSL hens, where the NaPi-IIb fragment expression correlated strongly with duodenal phosphatase activity (r = 0.47, *P* = 0.004), plasma Ca (r = 0.39, *P* = 0.018), estradiol (r = 0.57, *P* < 0.001), and PTH concentrations (r = - 0.50, *P* = 0.002). TRPV6 expression across both strains showed a positive correlation only with P concentration in the duodenum+jejunum (r = 0.26, *P* = 0.022). In LB hens, this correlation was stronger (r = 0.44, *P* = 0.006) and accompanied by negative correlations with the MI concentration in the duodenum+jejunum (r = - 0.38, *P* = 0.019), plasma Ca (r = - 0.36, *P* = 0.025), and calcitriol levels (r = - 0.40, *P* = 0.015). No significant correlations were observed for TRPV6 expression in LSL hens.

**Table 1:** Pearson correlation coefficients of the expression of NaPi-IIb fragment and TRPV6 in duodenal BBM with some systemic and local traits in two laying hen strains under dietary mineral P renunciation.

	Both strains, n=80 hens		LB, n=40 hens		LSL, n=40 hens	
	NaPi-IIb	TRPV6	NaPi-IIb	TRPV6	NaPi-IIb	TRPV6
Plasma P_i_^1^	0.05	0.05	0.09	0.15	0.03	-0.02
Plasma Ca^1^	0.12	-0.22	-0.12	-0.36*	0.39*	-0.06
Plasma MI^1^	-0.15	-0.03	-0.26	-0.13	0.03	0.15
D+J Ca^1^	-0.13	0.04	-0.09	0.04	-0.16	0.05
D+J P^1^	-0.06	0.26*	0.08	0.44⁎⁎	-0.20	0.08
D+J MI^1^	-0.13	-0.22	-0.14	-0.38*	-0.15	-0.02
D+J InsP_6_^1^	-0.06	0.11	-0.09	0.15	0.04	0.20
Phosph. act.^1^	0.36⁎⁎	-0.09	0.28	-0.04	0.47⁎⁎	-0.16
Calcidiol^2^	0.08	-0.01	0.30	-0.01	0.02	0.30
Calcitriol^2^	-0.11	-0.14	-0.15	-0.40*	-0.12	0.02
Estradiol^3^	0.25*	-0.14	-0.03	-0.25	0.57⁎⁎⁎	0.02
PTH^2^	-0.29*	-0.05	0.01	-0.12	-0.50⁎⁎	-0.01

⁎ *P* < 0.050,

⁎⁎ *P* < 0.010,

⁎⁎⁎ *P* < 0.001 indicate significant correlation coefficients. Abbreviations: LB = Lohmann Brown-Classic, LSL = Lohmann LSL-Classic, MI = *myo*-inositol, D+J = duodenum+jejunum, Phosph. act = duodenal phosphatase activity, PTH = parathyroid hormone.

1 Data from Sommerfeld et al., 2024.

2 Data from Qasir et al., 2025.

3 P-Fowl unit, unpublished data.

In the MI supplementation trial, the NaPi-IIb fragment and TRPV6 expression in LB hens were positively correlated with duodenal phosphatase activity (NaPi-IIb fragment: r = 0.32, *P* = 0.042; TRPV6: r = 0.45, *P* = 0.004; Table 2). In contrast, in LSL hens, NaPi-IIb fragment expression was negatively correlated with duodenal phosphatase activity (r = - 0.33, *P* = 0.041) and MI concentration in the duodenum+jejunum (r = - 0.37, *P* = 0.018). Additionally, TRPV6 expression in LSL hens showed a strong positive correlation with plasma calcidiol (r = 0.55, *P* < 0.001).

**Table 2:** Pearson correlation coefficients of the expression of NaPi-IIb fragment and TRPV6 in duodenal BBM with some systemic and local traits in two laying hen strains under dietary MI supplementation.

	Both strains, n=80 hens		LB n=40 hens		LSL, n=40 hens	
	NaPi-IIb	TRPV6	NaPi-IIb	TRPV6	NaPi-IIb	TRPV6
Plasma P_i_^1^	-0.12	-0.09	-0.02	0.15	-0.19	-0.21
Plasma Ca^1^	0.06	0.04	0.14	0.28	0.02	-0.06
Plasma MI^1^	-0.04	-0.01	0.20	0.20	-0.26	-0.09
D+J MI^1^	-0.08	-0.10	0.20	0.16	-0.37*	-0.24
Phosph. act.^1^	-0.02	-0.01	0.32*	0.45⁎⁎	-0.33*	-0.23
Calcidiol^2^	0.02	-0.02	0.14	-0.03	0.00	0.55⁎⁎⁎

⁎ *P* < 0.050,

⁎⁎ *P* < 0.010,

⁎⁎⁎ *P* < 0.001 indicate significant correlation coefficients. Abbreviations: LB = Lohmann Brown-Classic, LSL = Lohmann LSL-Classic, MI = *myo*-inositol, D+J = duodenum+jejunum, Phosph. act = duodenal phosphatase activity.

1 Data from Sommerfeld et al., 2025.

2 P-Fowl unit, unpublished data.

## Discussion

P and Ca homeostasis in laying hens is shaped by dietary supply, physiological stage, and genetic background. Current dietary P requirements of laying hens appear to be overestimated (Rodehutscord et al., 2023), and hens may tolerate markedly reduced dietary P without impairing mineral balance (Sommerfeld et al., 2020b). This provided the rationale for the mineral P renunciation trial, which examined whether hens can maintain mineral homeostasis during complete withdrawal of mineral P supplementation across two key physiological stages: pre-lay (wk 19) and onset of lay (wk 24), when mineral demands differ substantially. The physiological and mineral-regulatory outcomes of this trial are detailed in Sommerfeld et al. (2024) and Qasir et al. (2025). The MI supplementation trial was motivated by previously observed strain-dependent differences in MI metabolism between LB and LSL hens (Sommerfeld et al., 2020a; Gonzalez-Uarquin et al., 2021), prompting the evaluation of graded dietary MI inclusion in both genotypes. Given the antioxidant potential of MI (Gonzalez-Uarquin et al., 2020), the peak lay period (wk 26 - 30), was selected as a physiologically demanding stage characterized by elevated metabolic activity and oxidative stress (Wang et al., 2024). The metabolic and MI-related responses observed in the MI supplementation trial are described in Sommerfeld et al. (2025) and Szentgyörgyi et al. (2025).

Within this experimental framework, the current study focused on the intestinal BBM transporters NaPi-IIb and TRPV6, which represent the rate-limiting steps of active, transcellular P_i_ and Ca uptake and are highly expressed in chicken duodenum (Yan et al., 2007; Yang et al., 2011). Both dietary mineral P withdrawal and MI supplementation are known to influence luminal and systemic conditions relevant to mineral absorption (Sommerfeld et al., 2024; Sommerfeld et al., 2025; Qasir et al., 2025; Ogunribido et al., 2022), yet protein-level data on NaPi-IIb and TRPV6 expression under such conditions are largely lacking. Quantifying their abundance in the duodenal BBM therefore may provide essential complementary information on how intestinal P and Ca absorption potential may vary with diet, strain, and physiological stage in laying hens, an aspect directly investigated in this study.

### Interpretation of NaPi-IIb and TRPV6 Bands as detected by Western Blotting

In the present study, the anti-NaPi-IIb antibody revealed an immunoreactive band at approximately 45 kDa. The core molecular weight of the chicken NaPi-IIb transporter is approximately 74 kDa (Yan et al., 2007; Li et al., 2018), though it may range from 90 to 100 kDa due to variations in glycosylation (Arima et al., 2002). This variability is attributed to six potential N-linked glycosylation sites within a large putative extracellular loop (Yan et al., 2007). No study explicitly reported NaPi-IIb cleavage into low-molecular-weight fragments in avian or mammalian tissues; however, analogous post-translational processing was well characterized for the closely related renal NaPi-IIa cotransporter (Biber et al., 1996; Boyer et al., 1996; Xiao et al., 1997). Exposure of NaPi-IIa to reducing agents disrupted disulfide bonds within the large extracellular loop and generated two stable fragments of approximately 50 and 40 kDa, corresponding respectively to the N- and C-terminal domains of the same protein, which remained covalently linked under non-reducing conditions (Biber et al., 1996). This defined fragmentation pattern, which arose from reduction of disulfide-linked domains and was unaffected by protease inhibition, was interpreted as an intrinsic structural feature of NaPi type II cotransporter (Boyer et al., 1996; Xiao et al., 1997). In addition, Boyer et al. (1996) reported that both the intact and truncated NaPi-II proteins in rat renal BBM increased in response to a low-phosphate diet, indicating that the smaller NaPi-related fragment was a genuine product of the transporter. Research in human showed that variations in NaPi-IIb molecular weight could be induced by *SLC34A2* variants, resulting in truncated NaPi-IIb forms with molecular weight ∼ 35 kDa and ∼ 60 kDa along with a loss in their functionality (Jönsson et al., 2022). Given the conserved structural organization of *SLC34* transporters, including the presence of a large disulfide-linked extracellular loop shared by NaPi-IIa and NaPi-IIb (Wagner et al., 2014), the ∼ 45 kDa NaPi-IIb immunoreactive band observed in the present study most likely represented a truncated or reduced C-terminal fragment of the transporter formed through partial cleavage or disulfide-bond reduction. The complete disappearance of this band following peptide blocking further supported its specificity and confirmed that the signal originated from NaPi-IIb rather than from non-specific antibody binding.

Information on TRPV6 protein expression in the intestine of laying hens is limited. Most studies on intestinal Ca²⁺ absorption in chickens were focused on the intracellular transport and basolateral extrusion phases of the transcellular pathway (Bar, 2008; Li at al., 2018; Wang et al., 2022). This research gap may be attributed to inconsistencies in detecting TRPV6 mRNA expression in the chicken intestine, as reported in several studies (Proszkowiec-Weglarz et al., 2019; Garcia-Mejia et al., 2024). TRPV6 in laying hens was detected at ∼ 80 kDa across all intestinal sections, with the highest expression in the duodenum and the lowest in the rectum (Yang et al., 2011). In the present study, immunoreactive TRPV6 bands were observed at approximately ∼ 100 kDa in the duodenal BBM, consistent with previous findings (Huber et al., 2015). Although the specificity of the TRPV6 primary antibody was not directly verified in this experiment, the same antibody was previously validated for use in chickens through peptide-blocking assays in studies by Yang et al. (2011) and Huber et al. (2015), supporting the interpretation of detected band as TRPV6.

### The expression of NaPi-IIb fragment and TRPV6 in duodenal BBM under dietary mineral P renunciation as affected by age and strain

***Diet Effect.*** Dietary P reduction can trigger an adaptive response in the body, leading to adjustments in key transporter transcription and translation to compensate for the altered conditions. Previous studies in mice and broilers demonstrated that the body responded to a low-P diet by enhancing Na-P_i_ transport activity and increasing NaPi-IIb protein and mRNA levels (Hattenhauer et al., 1999; Fang et al., 2012). However, in laying hens, a low-P diet significantly reduced NaPi-IIb protein expression in the duodenum, while its expression in the jejunum and ileum remained unchanged, but NaPi-IIb mRNA expression was upregulated only in the ileum, highlighting a discrepancy between gene transcription and protein expression effects (Li et al., 2018). In the present study, dietary P levels influenced plasma P_i_ concentration, with lower values observed in groups without mineral P supplement (*P* < 0.001; Sommerfeld et al., 2024), but had no effect on the expression of NaPi-IIb fragment in the duodenum. Furthermore, the expression of the NaPi-IIb-encoding gene, *SLC34A2*, in the jejunum of the same hens was not affected by dietary mineral P renunciation for either strain (Abitew et al., 2024), consistent with findings by Jing et al. (2018). Beyond dietary P levels, luminal P_i_ availability for absorption in the gut may play a key role in modulating P_i_ transporter expression (Huber et al., 2015). In the present study, total P concentration was measured in the duodenum+jejunum (Sommerfeld et al., 2024), and it was not associated with the expression of NaPi-IIb fragment. However, the positive correlation between NaPi-IIb fragment expression level and duodenal phosphatase activity in both strains supports the notion that bioavailable P_i_ can modulate P_i_ transporter expression. Alternatively, this relationship may reflect a systemic effect, where greater P_i_ absorption capacity is associated with increased duodenal phosphatase activity, or vice versa, in a bidirectional manner.

Poultry studies have reported inconsistent findings regarding the intestinal adaptation of Ca absorption to low-P diets. While some studies observed decreased calbindin expression at both the mRNA and protein levels (Li et al., 2012; Nie et al., 2013), others noted increased mRNA expression of CaBP-D28k and PMCA1b without corresponding changes at the protein level (Li et al., 2018). In the present study, TRPV6 protein expression in the duodenum was not influenced by dietary mineral P renunciation, consistent with findings in broilers (Huber et al., 2015). The positive correlation between TRPV6 protein expression and intestinal P, along with its negative correlation with plasma Ca concentration in LB hens, suggested that LB hens may adjust TRPV6 expression in response to plasma Ca:P ratio fluctuations.

The absence of dietary mineral P renunciation effects on duodenal expression of NaPi-IIb fragment and TRPV6 in the present study aligns with the broader multi-organ pattern: regulation of P and Ca homeostasis in these hens was governed primarily by genotype and maturation stage rather than short-term dietary P withdrawal (Abitew et al., 2024; Qasir et al., 2025; Meier et al., 2025). It is also plausible that the primary adaptive response to reduced mineral P intake occurred in the kidney. This interpretation is supported by the renal transcriptome of LB hens, which showed upregulation of *SLC34A1* (NaPi-IIa) together with downregulation of *SLC34A2* (NaPi-IIb) under dietary mineral P renunciation, whereas LSL hens exhibited no significant renal changes in their expression (Qasir et al., 2025).

***Age Effect.*** With the transition to the egg-laying phase, mineral demand increases markedly. In our study, LSL hens showed a clear rise in the expression of NaPi-IIb fragment from wk 19 to wk 24, whereas LB hens maintained its stable expression across these stages. However, both LSL and LB hens involved in this study exhibited an increase in *SLC34A2* gene expression in the jejunum at the onset of egg laying (Abitew et al., 2024), suggesting that the observed differences might result from post-transcriptional regulation. In LB hens, the absence of changes in the expression of NaPi-IIb fragment aligns with the study by Wang et al. (2022), who observed a significant increase in *SLC34A2* mRNA expression from wk 20 to wk 28 in duodenum and jejunum of Hy-Line Brown hens, but no corresponding change at the protein level. In LSL hens, the expression of NaPi-IIb fragment positively correlated with duodenal phosphatase activity, plasma Ca, and estradiol levels, while negatively correlated with PTH. The positive correlation with plasma estradiol in the present study is consistent with previous research on female rats, where estrogen was shown to stimulate intestinal sodium-dependent P_i_ absorption, which was associated with increased *SLC34A2* mRNA and protein expression (Xu et al., 2003). As PTH did not directly regulate intestinal NaPi-IIb protein expression (Reyer et al., 2021; Abitew et al., 2024), the negative correlation observed in this study likely reflected context-dependent physiological adjustments associated with mineral homeostasis.

For TRPV6 expression, no age-related change was detected in LSL hens, whereas LB hens tended to reduce its expression with the onset of laying (wk 24). This potential decrease may reflect the suppression of active Ca absorption by high plasma Ca concentrations with the onset of egg laying, as indicated by the negative correlation between plasma Ca and TRPV6 expression in LB hens, which is consistent with the regulatory patterns described by Proszkowiec-Weglarz and Angel (2013).

***Strain Effect.*** Previous studies revealed that although LB and LSL hens had similar egg-laying performance, they exhibited marked differences in body weight, immunity, bone metabolism, and phytate degradation capacity (Habig et al., 2012; Sommerfeld et al., 2020b). Transcriptomic analyses also confirmed strain-specific differences: in LSL hens, Ca- and P-related traits were linked to distinct immune and signaling pathways, while in LB hens, they were associated with overlapping metabolic and hormonal pathways, indicating a more intertwined and stress-responsive regulatory pattern (Iqbal et al., 2021). In wk 24 of the present study, LSL hens tended for higher expression of NaPi-IIb fragment and TRPV6 than LB hens. Notably, LSL hens also showed generally higher P and Ca retention than LB hens, especially when P-deficient diets were fed (Sommerfeld et al., 2024). It is possible that LB hens may rely more on P_i_ absorption in distal intestinal segments, as indicated by higher *SLC20A1* and *SLC34A2* expression in the ileum compared to LSL hens at peak laying (Sommerfeld et al., 2020b). Additionally, higher concentrations of TiO_2_ in the duodenum and jejunum of LB hens suggested slower digesta passage (Sommerfeld et al., 2020b; Sommerfeld et al., 2024), resulting in prolonged mucosa - digesta contact time. From the other point, LB hens may have a limited capacity to adapt their intestinal transporter expression, which may contribute to a tenser metabolic condition consistent with metabolite profiling studies indicating higher levels of metabolic inflammation and oxidative stress in LB hens compared to LSL hens (Szentgyörgyi et al., 2025). Furthermore, in LB hens, the positive correlation between TRPV6 protein expression and intestinal P and the negative correlation with intestinal MI suggest that these two factors might be linked to Ca homeostasis of LB hens. A stimulatory effect of high-P diets on TRPV6 gene expression in the duodenum has been reported in mice (Katsumata et al., 2014) and pigs (Vötterl et al., 2023). Despite all these phenotypic differences between the two strains, the genetic foundations and possible heritability must be studied before the consequences for breeding activities can be evaluated. Such work is ongoing by the Research Unit P-Fowl, of which the present study is a part.

### Effect of dietary MI supplementation on the expression of NaPi-IIb fragment and TRPV6 in duodenal BBM

Dietary MI supplementation modulated the expression of the NaPi-IIb fragment in a clear strain-dependent manner. Notably, these changes occurred without alterations in plasma P_i_ concentrations or duodenal phosphatase activity (Sommerfeld et al., 2025), indicating that MI-induced adjustments in P_i_ transporter expression were not reflected at the systemic level. One plausible explanation is that MI may affect intracellular P_i_ turnover through its role in the synthesis of InsP and PP-InsPs, with the latter functioning as high-energy intracellular P_i_ reservoirs and regulators of P_i_ homeostasis (Saiardi, 2012; Gu et al., 2024). These molecules can transiently bind P_i_, buffer cytosolic P_i_ fluctuations, and contribute to ATP turnover and metabolic signalling (Szijgyarto et al., 2011). PP-InsPs accumulated when intracellular P_i_ supply was adequate and fell rapidly during P_i_ limitation, with their synthesis being tightly coupled to cellular bioenergetic status (Gu et al., 2024). In the present study, LB hens responded to dietary MI supplementation with an upregulation of the NaPi-IIb fragment, consistent with the notion that MI may increase intracellular demand for P_i_ through enhanced InsP and PP-InsPs synthesis. Earlier work also reported MI-induced increases in NaPi-IIb mRNA expression in enterocytes (Ogunribido et al., 2022). The positive association between NaPi-IIb fragment expression and duodenal phosphatase activity in LB hens suggested coordinated P_i_ uptake and P_i_ liberation within the proximal intestine. Moreover, LB hens showed higher plasma P_i_ and intermittently higher plasma MI (Sommerfeld et al., 2020a), together with higher MI intake, ileal MI flow, and postileal MI disappearance in the present trial compared with LSL hens (Sommerfeld et al., 2025). In contrast, LSL hens exhibited a downregulation of the NaPi-IIb fragment at higher MI intake, accompanied by negative correlations with both phosphatase activity and intestinal MI concentration. This pattern may reflect a feedback mechanism in LSL hens, whereby increased luminal or intracellular MI diminishes the requirement for additional P_i_ entry through NaPi-IIb. Nonetheless, differential regulation of NaPi-IIb along the intestinal tract cannot be excluded, emphasizing the need for segment-specific analyses under dietary MI supplementation.

The stimulatory effect of MI at 1 g/kg on NaPi-IIb fragment expression, although statistically confirmed only in LB hens, is a particularly relevant finding, identifying MI as a promising feed additive for strategies aimed at reducing mineral P supplementation in poultry diets to conserve finite resources and lower environmental P excretion; however, its physiological relevance and practical value still require further validation.

## Conclusions

Dietary mineral P renunciation did not affect duodenal expression of NaPi-IIb fragment and TRPV6 in either strain, suggesting that transporter-level adjustments were not required at this intestinal site to maintain mineral balance under reduced P supply. During the transition from pre-lay (wk 19) to onset of lay (wk 24), LB and LSL hens displayed distinct regulatory patterns: LSL hens increased the expression of NaPi-IIb fragment with unchanged TRPV6, whereas LB hens tended to downregulate TRPV6 with stable expression of NaPi-IIb fragment. Such divergence reflects inherent strain differences in adapting intestinal uptake pathways to rising mineral demands, reinforcing the importance of genotype in dietary P and Ca management. Dietary MI supplementation modulated the expression of NaPi-IIb fragment in a strain-dependent manner, indicating regulatory mechanisms that are not yet understood and warrant investigation.

## CRediT authorship contribution statement

**Nataliia Shomina:** Writing – review & editing, Writing – original draft, Validation, Methodology, Investigation, Formal analysis, Data curation. **Vera Sommerfeld:** Writing – review & editing, Validation, Supervision, Methodology, Investigation, Formal analysis, Data curation, Conceptualization. **Anna Hanauska:** Writing – review & editing, Validation, Methodology, Investigation, Data curation. **Michael Oster:** Writing – review & editing, Validation, Data curation. **Markus Rodehutscord:** Writing – review & editing, Validation, Supervision, Methodology, Funding acquisition, Conceptualization. **Korinna Huber:** Writing – review & editing, Validation, Supervision, Methodology, Funding acquisition, Data curation, Conceptualization.

## Disclosures

The authors declare the following financial interests/personal relationships which may be considered as potential competing interests:

Korinna Huber reports financial support was provided by German Research Foundation. Korinna Huber reports a relationship with German Research Foundation that includes: funding grants. If there are other authors, they declare that they have no known competing financial interests or personal relationships that could have appeared to influence the work reported in this paper.

## Appendix Supplementary materials

Image, application 1

## Footnotes

Supplementary material associated with this article can be found, in the online version, at doi:10.1016/j.psj.2025.106171.
