# Supplementary material for: Effects of dietary phosphorus and myo-inositol supplementation on NaPi-IIb and TRPV6 protein expression in duodenal apical membranes of laying hens from two strains
Source: Poult Sci. 2025 Nov 30;105(1):106171. doi: 10.1016/j.psj.2025.106171 (PMC12720333; doi:10.1016/j.psj.2025.106171)
Supplement: Supplementary file 1 [file mmc1.docx]

**Supplementary figure**

**
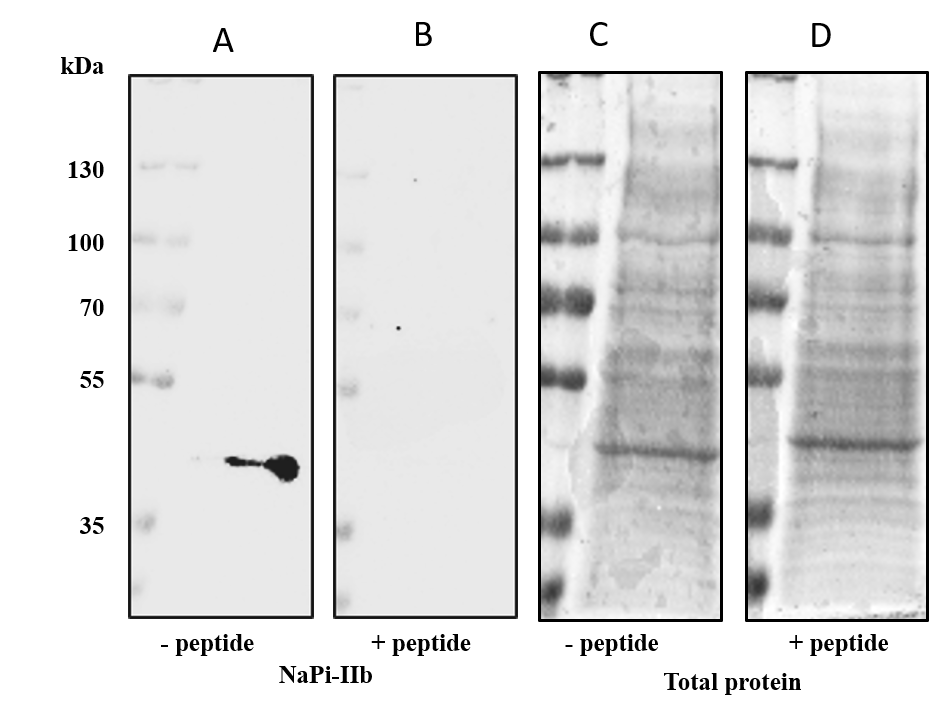
**

**Figure S1:** Validation of NaPi-IIb antibody specificity by blocking-peptide control.

Representative Western blots demonstrating specificity of the NaPi-IIb immunoreactive band (~45 kDa) in duodenal BBM preparations. **(A)** Immunoreactive ~45 kDa band detected when the primary antibody was used without blocking peptide (– peptide). **(B)** Complete loss of the ~45 kDa band after pre-incubation of the primary antibody with its antigenic blocking peptide (+ peptide), confirming specificity. **(C, D)** Indian ink total protein staining corresponding to panels A (C) and B (D), serving as loading controls.
